# Differences in duplication age distributions between human GPCRs and their downstream genes from a network prospective

**DOI:** 10.1186/1471-2164-10-S1-S14

**Published:** 2009-07-07

**Authors:** Yong Huang, Ying Zheng, Zhixi Su, Xun Gu

**Affiliations:** 1 Department of Genetics, Development, and Cell Biology, Center for Bioinformatics and Biological Statistics, Iowa State University, Ames, IA 50011, USA; 2 Institutes of Biomedical Sciences, School of Life Sciences, Center for Evolutionary Biology, Fudan University, Shanghai 200433, PR China

## Abstract

**Background:**

How gene duplication has influenced the evolution of gene networks is one of the core problems in evolution. Current duplication-divergence theories generally suggested that genes on the periphery of the networks were preferentially retained after gene duplication. However, previous studies were mostly based on gene networks in invertebrate species, and they had the inherent shortcoming of not being able to provide information on how the duplication-divergence process proceeded along the time axis during major speciation events.

**Results:**

In this study, we constructed a model system consisting of human G protein-coupled receptors (GPCRs) and their downstream genes in the GPCR pathways. These two groups of genes offered a natural partition of genes in the peripheral and the backbone layers of the network. Analysis of the age distributions of the duplication events in human GPCRs and "downstream genes" gene families indicated that they both experienced an explosive expansion at the time of early vertebrate emergence. However, we found only GPCR families saw a continued expansion after early vertebrates, mostly prominently in several small subfamilies of GPCRs involved in immune responses and sensory responses.

**Conclusion:**

In general, in the human GPCR model system, we found that the position of a gene in the gene networks has significant influences on the likelihood of fixation of its duplicates. However, for a super gene family, the influence was not uniform among subfamilies. For super families, such as GPCRs, whose gene basis of expression diversity was well established at early vertebrates, continued expansions were mostly prominent in particular small subfamilies mainly involved in lineage-specific functions.

## Background

Gene duplications at genomic and local levels are believed to have played important roles in the evolution of vertebrates [1-4]. Waves of gene duplication events were found to have happened at approximately the time of the emergence of early vertebrates and mammals [2]. Massive gene duplications would bring great disturbance to the gene regulatory networks in the cell. How gene duplications impacted and reshaped the gene networks was still not well understood. Nevertheless, several recent theoretical analyses have shed some light on the issue [5-8]. It was shown that the scale-free properties of the gene networks were necessary consequences under the assumption of asymmetric retention of duplicated genes in favor of the genes in the periphery of the network, which was supported by the family sizes of genes with different connectivity in genetic or protein-protein interaction (PPI) networks in yeast and worm [5,7].

However, these studies did not provide information as to how the duplication-divergence process [5] proceeded along the time axis during major speciation events, such as the emergence of vertebrates, as their model species were all invertebrates. Meanwhile, the genetic or PPI networks offered only snapshot information about the relationship between family sizes and connectivity of genes, which was often found to be inaccurate. Independent evidences not directly based on genetic or PPI networks were needed for cross examination.

In view of these problems, in this study, we used human G-protein coupled receptors (GPCRs) and their downstream genes in the pathways ("downstream genes") as the model system to examine the impact of gene duplication on the evolution of genes in different layers of the network. It has been shown that the gene regulatory network roughly maps to the cellular organization, with the genes on the periphery of the cell maps to the peripheral layer of the gene network [9]. In this sense, GPCRs and their "downstream genes" offered a natural partition of the peripheral layer and the backbone layer of the gene network. Meanwhile, GPCRs also form one of the largest known groups of signaling proteins in mammalian genomes [10], and GPCR pathways cover a good portion of the gene network and influence a wide range of physiological activities such as neurotransmission, metabolism, secretion, differentiation and growth, learning and memory, and immune responses [11-13]. The results from the GPCR model system were thus highly representative of the general gene regulatory network in human cells.

In this study, we estimated the ages of the duplication events in human GPCRs and the "downstream genes" gene families. Comparison of the age distributions of GPCRs *vs. *the "downstream genes" provided a more detailed view of the duplication-divergence process along the time axis in the context of major speciation events in vertebrates. Furthermore, GPCRs were partitioned according to the GRAFS system [14] into subfamilies, and the age distributions of major subfamilies of GPCRs were estimated and compared. We also examined the expression profiles of GPCRs and downstream genes of different duplication ages, for their contribution to the tissue complexity at different evolutionary stages. In general, we found that most of the GPCR pathways, which cover a substantial portion of the gene network, have been established at the time of early vertebrate emergence. Continued expansions in GPCR families were to a large extent contributed by several small subfamilies involved in immune responses and sensory responses. Our study of the GPCR pathways suggested that the position of a gene in the gene network has great influence on the likelihood of fixation of its duplicates. However, the influence was not uniform. Instead, expansion of a large gene family may be attributed to strong expansions of some particular subfamilies, when it was favorable in particular species, or at particular evolutionary stages. The generality of these principles could be further examined in other super gene families when the data become available.

## Results

### Defining the model system of GPCRs and the "downstream genes"

**Figure 1:**
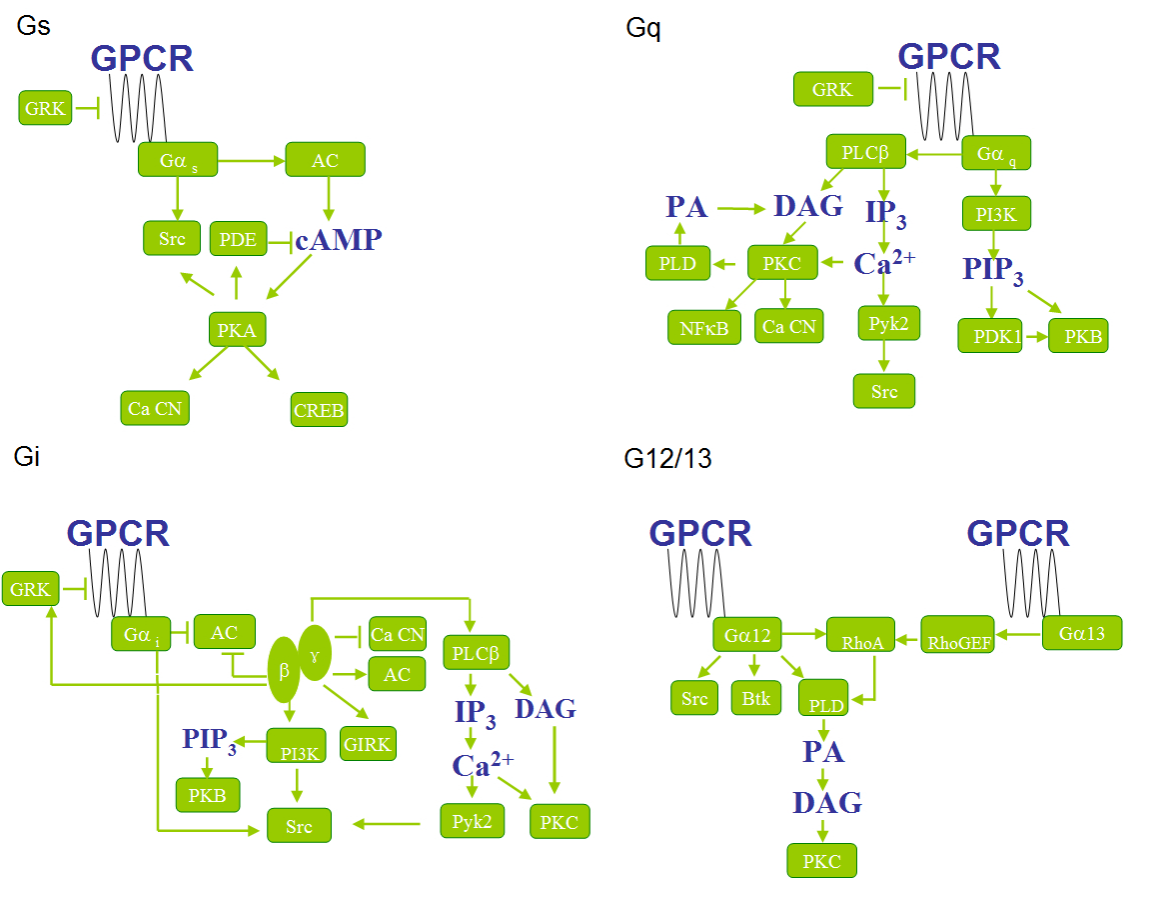
**Major GPCR pathways, G_s_, G_i/o_, G_q/11_, G_12/13_**.

Our model system of human GPCRs and the "downstream genes" was based on the classical GPCR signal pathways, which has been summarized in [15]. Typically, a stimulated GPCR activates one or more types of G proteins (the transducers) which further activate specific effectors of various downstream pathways. G Proteins are composed of an α-subunit and a βγ-subunit (heterodimer stable at physiological conditions). The specificity of the cellular downstream pathways of GPCRs is largely determined by the type of Gα subunits it binds. There are four major subfamilies for Gα, including Gα_s_, Gα_i/o_, Gα_q/11_, and Gα_12/13_, which define the corresponding GPCR-related signaling pathways: G_s_, G_i/o_, G_q/11_, G_12/13 _and G_βγ _pathways. We included the major genes in these pathways from the summary [15] as the "downstream genes", including the G proteins but excluding any genes that were cross-talking receptors or ion-channels on the cell membrane. The "downstream genes" in the pathways were summarized in Figure 1. The G_βγ _pathway was not specifically shown as all its downstream genes were covered in the G_i/o_pathway already. Vertebrate and invertebrate homologues of the human "downstream genes" were identified and added to the families in order to reconstruct the phylogenetic trees and estimate of the ages of the duplication events (see Methods).

We studied the human non-olfactory GPCRs in this study which were classified based on the GRAFS classification system [14]. The olfactory GPCRs were not included in this study, as their evolutionary patterns in mammals was peculiar [16]. We also excluded 23 human non-olfactory GPCRs that were not classified by the GRAFS system [14], 11 GPCRs in the delta group of Rhodopsin that were likely misclassified, and several predicted GPCR genes that were no longer supported by current genome annotations. In total, our GPCR data set covered 52 human GPCR families containing 302 human non-olfactory GPCRs. Vertebrate and invertebrate homologues of the human GPCRs were identified and linked to the families (see Methods).

### Estimating the duplication ages of human GPCRs and the "downstream genes"

**Figure 2:**
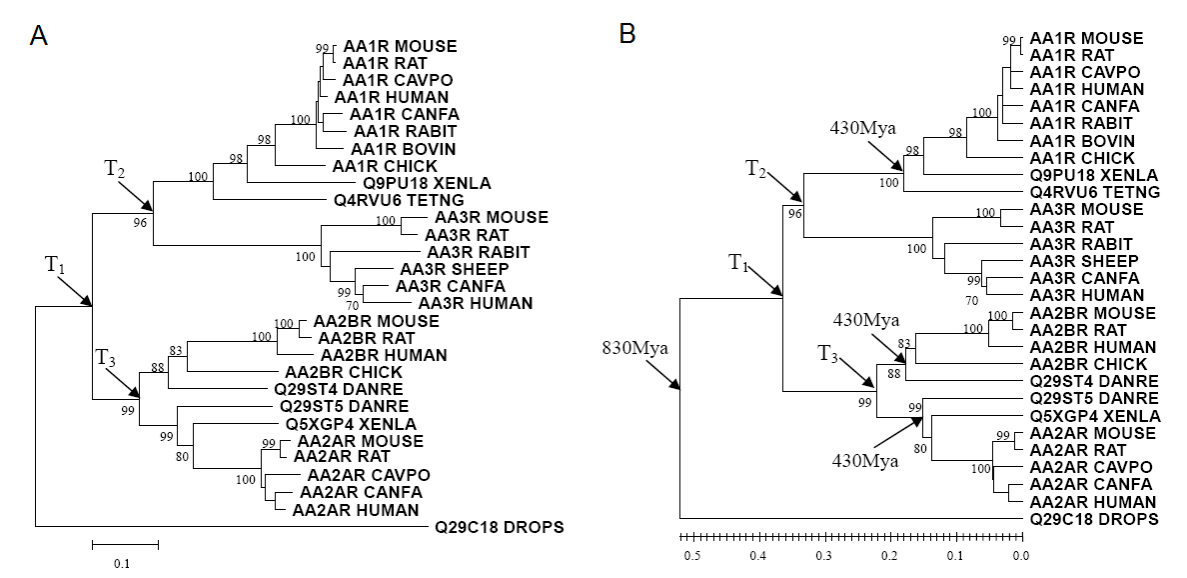
**An example of how the ages of duplication events were estimated in the ADORA family**. The neighbor-Joining phylogenetic tree (A) was constructed based on the protein sequences, and the correspondent linearized tree (B) was also calculated. The protein sequence distances (mutation per amino acid residue) were marked on the graph as the scales. The duplication events, T1–T3, were marked on the trees, and the age of the duplication events were estimated based on the linearized trees (see Methods).

We estimated the ages of the duplication events of human GPCRs and "downstream genes" using the "nearest neighbor clock" approach we introduced in a previous work [2]. Briefly, a phylogenetic tree was constructed for each family. The age of a divergence event was estimated based on its distances to the nearest bracketing species-split times in the phylogenetic tree under the molecular clock hypothesis [17,18]. Shown in Figure 2 was an example of the process for the Adenosine Receptor A subfamily (ADORA), which is a subfamily in the alpha group of the Rhodopsin class. The ADORA subfamily has four human genes and 25 homologous sequences from other species as distant as fly. We first reconstructed the phylogenetic tree (Neighbor-Joining) based on the protein sequences and calculated the linearized tree [18]. Among the four human genes in the ADORA family, there were three duplication events T1–T3 (always one less than the number of human genes, in bifurcate trees), which were marked in the phylogenetic tree in Figure 2. For T3 as an example, the nearest bracketing species-split times were 830 Myrs (million years before present, same below) of the vertebrate-fly split and 430 Myrs of the tetrapod-teleost split. The age of T3 should satisfy (830-T3)/(T3-430) = d1/d2, where d1 was the distance from T3 to the vertebrate-fly split and d2 was the average distance from T3 to the two subordinate tetrapod-teleost splits in the linearized tree. Distances d1 and d2 were directly measurable from the linearized tree. T3 was thus estimated as 487.95 Myrs. The ages of other duplication events were calculated likewise. The duplication ages of all the GPCRs in our dataset was summarized in Additional file 1.

For the "downstream genes", the process was essentially the same. The ages were estimated using the "nearest neighbor clock" approach as well for each family and were summarized in Additional file 2.

### Differences in age distribution between GPCRs and the "downstream genes"

**Figure 3:**
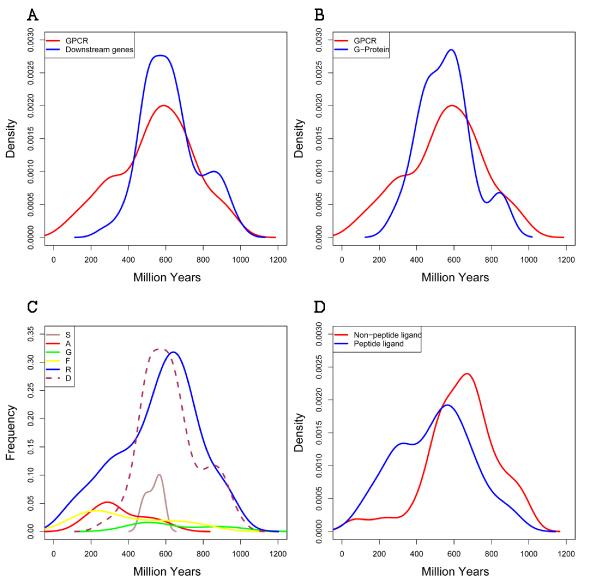
**Comparison of duplication age distributions between different groups of genes**. The x-axis was the evolutionary age (from now) in million years. The y-axis was the density of the distributions. A) GPCRs *vs. *the "downstream genes"; B) GPCRs *vs. *G Proteins; C) Between different subclasses of GPCRs (The densities was multiplied by the number of genes in correspondent subclasses to reflect the differences in frequencies); D) GPCRs with peptide-ligands *vs. *non-peptide ligands.

We first compared the age distributions of GPCRs and the "downstream genes" in general. We focused on the divergence events happened after 1000 Myrs (before present, same below), as we were mainly interested in the evolution of vertebrates. Single homologues in fly were identified in most of the families, suggesting most of the gene families emerged early in evolution and stayed in single copy in the genomes up at least to the fly. As was shown in Figure 3A, however, both GPCRs and the "downstream genes" families had an explosive increase in duplication events which caused strong expansions of the families during the period of about 400–800 Myrs, peaked at around 600 Myrs. This was a time close to the emergence of early vertebrates. This pattern recaptured the general pattern of human gene families expansion that has been shown in one of our previous studies [2]. We have suggested that this rapid increase in paralogous genes at the early stage of vertebrate evolution were likely caused by genome duplications. However, the age distributions of GPCRs and the "downstream genes" differed greatly after 400 Myrs. Continued expansion of gene families was only observed for the GPCRs, while few families of "downstream genes" saw expansion after 400 Myrs. We further examined the age distributions of GPCRs and their direct downstream partners, G proteins. As was shown in Figure 3B, the age distributions of G proteins were close to the "downstream genes" in general, which also saw few duplication events after 400 Myrs. Taken together, we found that GPCRs families did experience more expansion than the "downstream genes" in more recent evolutionary stages when most gene duplications have been shown to be local small-scale duplication events [2]. However, at the time of early vertebrate emergence, when large scale genome duplication happened, both GPCRs and the "downstream genes" experienced similar explosive expansions.

We further examined the age distribution of duplication events of different subclasses of GPCRs to see if they contributed equally to the age distribution of GPCRs. Shown in Figure 3C were the age distributions of the GRAFS subclasses of GPCRs (and the "downstream genes (D)" in a dashed line as reference). The densities were multiplied by the number of genes in each class to reflect the differences in sizes among classes. Different classes of GPCRs contributed differently to the overall distribution of GPCRs. Rhodopsins, which makes up the majority of GPCRs, had a similar age distribution to GPCRs overall, with the peak slightly moved right to over 600 Myrs. The Secretin and Glutamate receptor classes had only an obvious expansion between 400 to 600 Myrs. In contrast, duplication events in the Adhesion and Frizzled/Taste2 receptor classes happened more recently and were mostly after 300 Myrs. Actually, the continued expansion of GPCR families after 400 Myrs were largely contributed by several small classes such as Adhesion and Frizzled/Taste2 receptors and the chemokine receptors of the Rhodopsin class (see Additional file 1 for the actually ages). This result showed that within the GPCR superfamily, and even within the Rhodopsin subclass, the expansion of different subfamilies was asymmetric.

In a recent report [19], GPCRs with non-peptide ligands were reported to have a significantly higher retention rate than GPCRs with peptide ligands after a lineage-specific whole genome duplications in the pufferfish *Tetraodon nigroviridis *more than 230 Myrs ago. We examined if the same was true for GPCRs in general. Based on the report [19], GPCRs that bind non-peptide ligands included GPCRs in the Glutamate receptor class and A1-A4 subclasses of the Rhodopsin class (see Additional file 1), while the rest GPCRs bind peptide ligands. As was shown in Figure 3D, it was obvious that more duplication events were found among the GPCRs that bind peptide ligands than those bind non-peptide ligands in more recent evolutionary stages (after 400 Myrs). This was in direct contrast to the results of the lineage-specific GPCRs in pufferfish by the report [19]. This may have reflected that the selective pressure driving the fixation of different types of duplicated GPCRs were different in different species and at different evolutionary stages. Actually, it was also shown in Figure 3D that, before 600 Myrs, more duplicated events were observed in GPCRs with non-peptide ligands than GPCRS with peptide-ligands. This was not anti-intuitive as many of the ancestor species emerged at that evolutionary stage were simple marine invertebrates.

In general, our results have shown that more duplication events were found for the GPCRs than the "downstream genes", particularly in more recent evolutionary stages when the duplication events were mostly local. This was consistent with the current theory of gene duplication and gene network. However, we have found several aspects that have not been covered in the current theory. First, at the time of emergence of early vertebrate, both GPCRs and the "downstream genes" families experienced explosive expansion. The asymmetric duplication-divergence process may be a good model for gene duplication at normal times, but more factors were likely to be in play at that evolutionary stage when massive genomic duplications and explosive increase in tissues complexity happened. Second, the expansion of gene families in the peripheral layer of the gene network may also be asymmetric. Certain small branches of the big family may get disproportional expansion in particular species at particular evolutionary stages, as exemplified by the type 2 taste receptors in human.

### Tissue distribution of GPCRs and the "downstream genes" of different duplication ages

**Figure 4:**
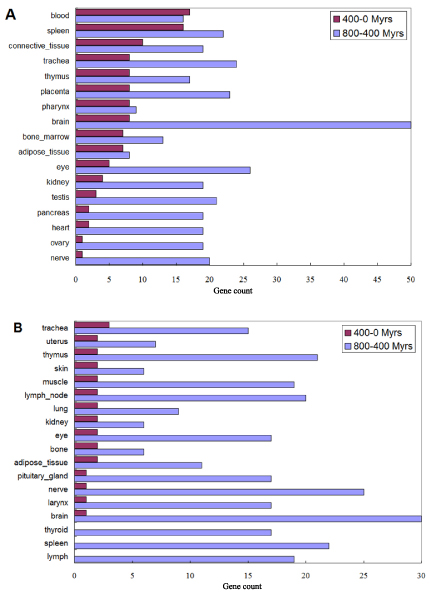
**Tissue expression distribution for GPCRs and "downstream genes"**. The numbers of genes that were highly expressed in the tissues and duplicated in different evolutionary time intervals were charted for GPCRs (A) and "downstream genes" (B).

Duplicated genes often have divergent expression patterns in tissues. We further examined the expression profiles of human GPCRs and the "downstream genes" with respect to their duplication ages. The expression profiles were based on the EST count information from UniGene [20]. For each human gene in the GPCR model system, we identified the tissues where it was highly expressed (see Methods for details). The duplication age of a human gene was defined as the age of the most recent duplication event leading to the gene. Due to the approximate nature of age estimation, we divided the duplication time into three broad intervals, including earlier than 800 Myrs, 800–400 Myrs and 400-0 Myrs, and focused on the last two intervals. For genes that duplicated in each of the intervals, and then for each of the 45 tissues covered by UniGene, we calculated the number of genes with high expression in that particular tissue. The result was summarized in Figure 4 for the top tissues in number of highly expressed genes of the GPCR model system.

Shown in Figure 4A was the result for GPCRs. Genes duplicated in the 400-0 Myrs interval were denoted as group 400, and genes duplicated in the 800-400 Myrs interval were denoted as group 800. The tissues were ranked by the number of group 400 genes that were highly expressed in a tissue. Our result showed that expression of the more recently duplicated genes (group 400) was enriched in blood and spleen, both of which important tissue for immune response. The chemokine receptor family contributed greatly to these two tissues. Enrichment of expression in the connective tissue was also observed, which was connected to the adhesion receptor family. On the other hand, group 800 genes were expressed in a much wider range of tissues. Interesting, most of the GPCRs expressed in the brain were in group 800. Relatively few GPCR genes expressed in brain or nerve tissues were recently duplicated.

Shown in Figure 4B was the result for the "downstream genes". The data series and the ranking of the tissues were defined the same as in Figure 4A. As has been shown earlier, relatively fewer "downstream genes" were duplication after 400 Myrs, which was reflected in the small number of genes in group 400 in Figure 4B. Similar to GPCRs, the "downstream genes" in group 800 were expressed in a wider range of tissues. Expression in brain and nerve again was most enriched in group 800.

In general, these results of tissue distribution indicated that a substantial enrichment of both GPCRs and the "downstream genes" expressed in brain and nerve tissues happened during the 800-400 Myrs interval. However, similar surge were not observed during the 400-0 Myrs interval. Instead, an enrichment of the GPCRs that expressed in the immune-related tissues of blood and spleen were observed, and this again was contributed mostly by several small GPCRs subfamilies.

## Discussion

Current theories of gene duplication and gene networks suggest that genes on the periphery of the network are preferentially retained after gene duplication, in comparison to the genes form the backbones of the network. This is actually necessary for the gene network to remain scale-free after rounds of gene duplication. However, the data supporting these theories were mostly based on the genetic network or the PPI network in yeast and worm. Similar data on gene networks in more advanced species, including vertebrates, were not yet complete and reliable enough. Our model system of GPCRs and the "downstream genes", took the advantage of the knowledge that the gene network roughly maps to the cellular organization [9], offered an opportunity to get some insight into the relationship of gene duplication and gene network in the context of vertebrate evolution. Our result showed that GPCRs families had significantly more continued expansion after 400 Myrs, in comparison to the "downstream genes". Under the assumption that all genes have equal opportunities for duplication, this result confirmed that duplicated GPCR genes were preferentially fixed during the 400-0 Myrs interval, compared to the "downstream genes". However, this preferential retention was time dependent, as during the 800-400 Myrs interval we found both GPCR and "downstream gene" families experienced explosive expansion. One explanation for the result of the 800-400 Myrs interval might be that tissue complexity of the species might also experience an explosive increase during that interval, which might have driven fixation of duplicated genes in all the layer of the network. The expression profiles of the genes with duplication ages in the 800-400 Myrs interval offered partial support for the explanation, as genes duplicated at that stage were found to be expressed broadly in a wide range of tissues in human, including brain and nerve. Our results suggested that the gene basis of tissue diversity was largely established by gene duplications in the 800-400 Myrs interval.

There may also be a species-dependence influencing the preference of fixation of duplicated genes within GPCRs. This was reflected in the difference in the preference of retention of GPCRs of peptide ligands *vs. *non-peptide ligands in human *vs. *in fish [19]. In fish, the retention rate of GPCRs of non-peptide ligands was higher than that of GPCRs of peptide ligands after a whole genome duplication 230 Myrs ago. In contrast, our results showed that, among human GPCRs, more GPCRs that bind peptide ligands were fixed after 400 Myrs than GPCRs of non-peptide ligands. In human, the subfamilies that contributed most to the continued expansion of GPCRs after 400 Myrs were those involved in immune responses and sensory responses. This may have reflected differences in environmental influences between human and fish on the selective pressure that drove the fixation of GPCR duplicates.

In this study, we have kept the model system simple by including only the downstream genes in the classical GPCR pathways. Many other genes that were indirectly influenced by GPCRs were not included in the study, which are mostly kinases and transcription factors in the signaling pathways. However, one of our previous studies [21] on the human tyrosin kinase super family found similar patterns with the "downstream genes".

## Conclusion

In general, using the human GPCR model system, our results confirmed that the position of a gene in the gene networks has great influences on the likelihood of expansion of its gene family in evolution. However, we also found that the influence was asymmetric among subfamilies of GPCRs. We found that the gene basis of expression diversity of most GPCR pathways, which cover a substantial portion of the gene network, have been established at the time of early vertebrate emergence. Continued expansions in human GPCR families were to a large extent contributed by several small subfamilies involved in immune responses and sensory responses in human. Exactly which subfamilies may see extra expansion may be contingent on environmental factors for different species at different evolutionary stages, as was exemplified by our comparison of the differences in retention preference of GPCRs binding peptide or non-peptide ligands between humans and fishes.

In the future, we shall further investigate the association between gene family expansions and GPCR-related networks [22-27]. For instance, we may study the functional divergence between the duplicated GPCR-related proteins [22,23], the role of alternative splicing isoforms [24], as well as the tissue-specific effects [25,26] on the gene evolution. Recently, Gu [27] proposed an evolutionary model for the origin of modularity in a complex gene network, suggesting that new (protein-protein) interactions after the gene duplication may be favored to link preexisted backbone of signaling pathway, while loss of interactions are at random. The vertebrate GPCR networks may be an ideal model to study the design principle of signaling networks.

## Materials and methods

### Family and sequence data

The GPCR gene families analyzed in this study were classified based on the GRAFS classification system [14], which covers 342 human non-olfactory GPCRs in five major classes including Glutamate (15), Rhodopsin (241), Adhesion (24), Frizzled/taste2 (24), Secretin (15). The Rhodopsin class is further divided into four groups: alpha, beta, gamma and delta. The sequences of vertebrate GPCR genes were retrieved from the Hovergen database [28]. In cases where the GRAFS families were further split in Hovergen, the split families were used for age estimation separately. The invertebrate homologues, as well as some vertebrate homologues missed by Hovergen, were identified by extensive BLAST [29] searching of the Swiss-prot protein database [30]. Redundant sequences were removed either based on UniGene annotations when available, or based on chromosome positions otherwise.

The "downstream genes" families were constructed similarly. Families were constructed for each entry in Figure 1. The vertebrate homologues in the gene families were retrieved from Hovergen [28], the invertebrate homologues and the vertebrate homolgues missed by Hovergen were identified by homology searching in the Swiss-prot database. Redundant sequences were removed similarly as for GPCRs.

### Inference of duplication events and estimating their ages

In bifurcate trees, each new human homologue is created by a duplication event. As a result, for a gene family with n human genes, there will be n-1 duplication events. We used the "nearest-neighbor clock" approach [2] to date the duplication events. For a gene family, we first reconstructed the phylogenetic tree. We used Clustal W [31] to carry out multiple sequence alignment of the protein sequences of the genes in the family. Phylogenetic trees (Neighbour-joining) were reconstructed using the MEGA program [32]. Linearized trees [18] were also calculated using MEGA. Next, the age of a duplication event was estimated based on its distances to the nearest bracketing species-split times in the phylogenetic tree under the molecular clock hypothesis [17,18]. We used the widely accepted species split times for calibration, including primate-rodent (80 Myrs ago), mammal-bird (310 Myrs ago), mammal-amphibian (350 Myr ago), tetrapod-teleost (430 Myr ago) and vertebrate-fly splits (830 Myr ago) [33]. In cases that a bracketing pair of species-split times can be found for a duplication event, the age of the event was just the linear interpolation based on the distances of the event in the linearized tree to the species-split times. In cases that no bracketing species-split times can be found for a duplication event (usually for very ancient or recent duplication events), linear extrapolation was used to calculate the age.

### Expression profiles of human genes by UniGene

We used the EST counts in UniGene [20] as the expression profiles of the human genes in the tissues. The ratio of the number of EST clones of a gene vs. the total number of clone in the library in a tissue was treated as the expression level of the gene in the tissues. For each gene, we calculated its mean expression level and the standard deviation across the 45 tissues covered in UniGene. The distribution of the expression levels of a gene across species generally fit a gamma distribution with small mean and large variation. We defined a gene highly expressed in a tissue if its expression level was one standard deviation greater than the mean expression level.

## Competing interests

The authors declare that they have no competing interests.

## Authors' contributions

XG, ZS, YZ and YH conceived the study. XG coordinated the project and the manuscript preparation. YH, YZ and ZS carried out the data analysis and drafted the manuscript. XG and ZS coordinated the project and the manuscript preparation. All authors read and approved the final manuscript.

## Supplementary Material

Additional file 1 Duplication age distribution of GPCR genes. Click here for file

Additional file 2 Duplication age distribution of "downstream genes". Click here for file
